# Supplementary material for: Mutations in PCYT2 disrupt etherlipid biosynthesis and cause a complex hereditary spastic paraplegia
Source: Brain. 2019 Oct 22;142(11):3382–97. doi: 10.1093/brain/awz291 (PMC6821184; doi:10.1093/brain/awz291)
Supplement: awz291_Supplementary_Data [file awz291_supplementary_data.zip › awz291-Suppl_data/Supplementary_Data4.zip!/Supplementary information 3 - fibroblast lipidomics.zip!/Fibroblast lipidomics/Controle vs Patient1/Statistical plots and data/PC species/Heatmap.pdf]

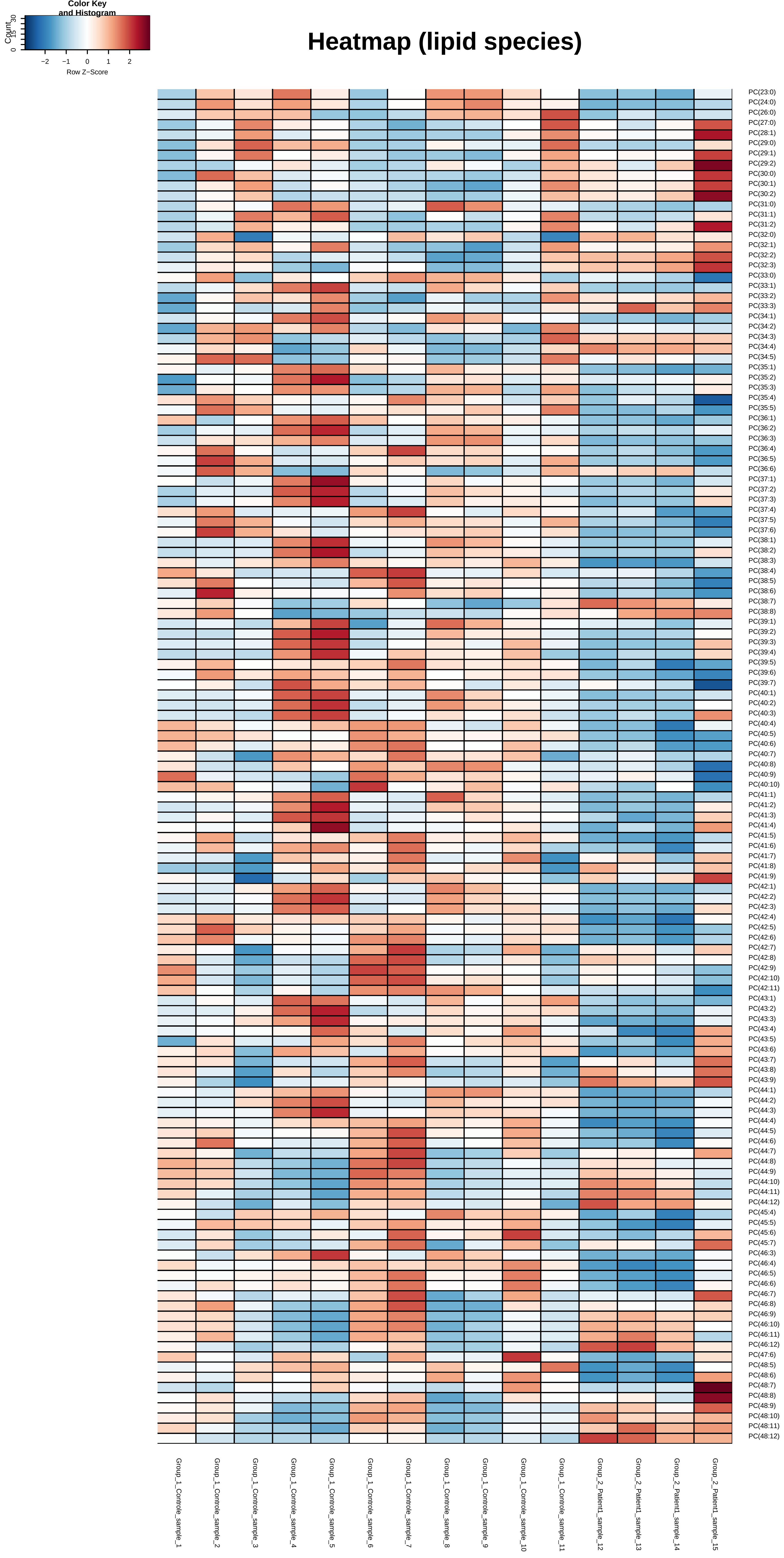
Color Key
and Histogram
Count
0
15
30
−2
−1
0
1
2
Row Z−Score
Heatmap (lipid species)
PC(23:0)
PC(24:0)
PC(26:0)
PC(27:0)
PC(28:1)
PC(29:0)
PC(29:1)
PC(29:2)
PC(30:0)
PC(30:1)
PC(30:2)
PC(31:0)
PC(31:1)
PC(31:2)
PC(32:0)
PC(32:1)
PC(32:2)
PC(32:3)
PC(33:0)
PC(33:1)
PC(33:2)
PC(33:3)
PC(34:1)
PC(34:2)
PC(34:3)
PC(34:4)
PC(34:5)
PC(35:1)
PC(35:2)
PC(35:3)
PC(35:4)
PC(35:5)
PC(36:1)
PC(36:2)
PC(36:3)
PC(36:4)
PC(36:5)
PC(36:6)
PC(37:1)
PC(37:2)
PC(37:3)
PC(37:4)
PC(37:5)
PC(37:6)
PC(38:1)
PC(38:2)
PC(38:3)
PC(38:4)
PC(38:5)
PC(38:6)
PC(38:7)
PC(38:8)
PC(39:1)
PC(39:2)
PC(39:3)
PC(39:4)
PC(39:5)
PC(39:6)
PC(39:7)
PC(40:1)
PC(40:2)
PC(40:3)
PC(40:4)
PC(40:5)
PC(40:6)
PC(40:7)
PC(40:8)
PC(40:9)
PC(40:10)
PC(41:1)
PC(41:2)
PC(41:3)
PC(41:4)
PC(41:5)
PC(41:6)
PC(41:7)
PC(41:8)
PC(41:9)
PC(42:1)
PC(42:2)
PC(42:3)
PC(42:4)
PC(42:5)
PC(42:6)
PC(42:7)
PC(42:8)
PC(42:9)
PC(42:10)
PC(42:11)
PC(43:1)
PC(43:2)
PC(43:3)
PC(43:4)
PC(43:5)
PC(43:6)
PC(43:7)
PC(43:8)
PC(43:9)
PC(44:1)
PC(44:2)
PC(44:3)
PC(44:4)
PC(44:5)
PC(44:6)
PC(44:7)
PC(44:8)
PC(44:9)
PC(44:10)
PC(44:11)
PC(44:12)
PC(45:4)
PC(45:5)
PC(45:6)
PC(45:7)
PC(46:3)
PC(46:4)
PC(46:5)
PC(46:6)
PC(46:7)
PC(46:8)
PC(46:9)
PC(46:10)
PC(46:11)
PC(46:12)
PC(47:6)
PC(48:5)
PC(48:6)
PC(48:7)
PC(48:8)
PC(48:9)
PC(48:10)
PC(48:11)
PC(48:12)
Group_1_Controle_sample_1
Group_1_Controle_sample_2
Group_1_Controle_sample_3
Group_1_Controle_sample_4
Group_1_Controle_sample_5
Group_1_Controle_sample_6
Group_1_Controle_sample_7
Group_1_Controle_sample_8
Group_1_Controle_sample_9
Group_1_Controle_sample_10
Group_1_Controle_sample_11
Group_2_Patient1_sample_12
Group_2_Patient1_sample_13
Group_2_Patient1_sample_14
Group_2_Patient1_sample_15
